# Hepatoprotective and Hypolipidemic Effects of *Satureja Khuzestanica* Essential Oil in Alloxan-induced Type 1 Diabetic Rats 

**Published:** 2012

**Authors:** Hassan Ahmadvand, Majid Tavafi, Ali Reza Khalatbary

**Affiliations:** a *Department of Biochemistry, Faculty of Medicine, Lorestan University of Medical Sciences, Khorramabad, Iran. *; b *Razi Herbal Researches Center, Lorestan University of Medical Sciences, Khorramabad, Iran. *; c *Department of Anatomy, Faculty of Medicine, Lorestan University of Medical Sciences, Khorramabad, Iran. *; d *Department of Anatomy, School of Medicine, Mazandaran University of Medical Sciences, Khazar Boulevard, Sari, Iran*.

**Keywords:** Diabetes, Lipid profile, Atherogenic index, Rat, Liver. Enzymes, Total antioxidant

## Abstract

In the present study, we examined the antioxidative activities of *Satureja khuzestanica *essential oil (SKE) and possible protective effect of SKE on lipid profile, atherogenic index and liver enzyme markers in Alloxan-induced Type 1 diabetic rats. Thirty male rats were randomly divided into three groups; group one as control, group two diabetic untreatment, and group three treatments with SKE by 500 ppm in drinking water, respectively. Diabetes was induced in the second and third groups by alloxan injection subcutaneously. After 8 weeks, the levels of fasting blood glucose (FBG), triglyceride (TG), cholesterol (C), low density lipoprotein (LDL), very low density lipoprotein (VLDL), high density lipoprotein (HDL), atherogenic index and the activities of alanine aminotransferase (ALT), aspartate aminotransferase (AST) and alkaline phosphatase (ALP) of all groups were analyzed. Data were analyzed through non-parametric Man Whitney test (using SPSS 13 software) and p < 0.05 was considered significant. SKE inhibited significantly the activities of ALT and ALP and decrease FBG, TG, C, LDL and VLDL. HDL level was significantly increased when treated with the extract. The activities of AST stayed unaltered. Moreover, total antioxidant capacity of SKE was 3.20 ± 0.40 nmol of ascorbic acid equivalents/g SKE. This study showed that SKE is a source of potent antioxidants. The findings of the present study also suggest that SKE exert beneficial effects on the lipid profile, atherogenic index and liver enzymes activity in Alloxan-induced Type 1 diabetic rats.

## Introduction

Diabetes mellitus, one of the leading metabolic syndromes, accounts for highest morbidity and mortality worldwide (1). Diabetes mellitus is characterized by abnormalities in carbohydrate, lipid and protein metabolism due to complete or relative insufficiency of insulin secretion from pancreatic *β*-cells and/or defect in insulin action (2). Oxidative stress is a state of imbalance between oxidant and antioxidant systems (3). 

Recently, much attention has been focused on the central and key role of oxidative stress in the pathogenesis of different diabetic complications (4). Several studies have shown that antioxidant treatment reduces diabetic complications (5). 

Due to increasing demand of patients for the use of natural products and other herbal drugs with anti-diabetic activity, the general trend now is to use the natural products for medicinal application in their natural available form (6). Polyphenols, well-known antioxidants, have also proved to function as antidiabetic by reducing blood glucose levels (7-9). Researchers have become recently interested in investigation and research into extraction of natural antioxidants from medical herbs to replace synthetic antioxidants (10-13). Therefore, the research into the determination of the natural antioxidant source is very important to promote public health (12).

*Satureja-khuzestanica, *an endemic plant of Iran, decreases glucose and malondealdehyde in serum diabetic patients (14-16). The components of this extract were analyzed with gas chromatography/mass spectrometry (GC/MS) in Research Center of Lorestan University as reported in our previous paper (16). The main component of this extract is carvacrol as a good antioxidant (14). Since the hypolipidemic, antiatherogenic and liver protective effects of SKE have not previously been reported; the objectives of the present study were to investigate hepatoprotective, hypolipdemic and antiatherogenic effects of *Satureja khuzestanica *essential oil in Alloxan-induced Type 1 diabetic rats.

## Experimental

*Isolation of the essential oil from Satureja khuzestanica*

*Satureja khuzestanica *essential oil was prepared from cultivated *satureja khuzestanica *in Khorramabad (Lorestan province, western Iran). The aerial parts of the plants were collected during flowering stage and were air-dried at ambient temperature in the shade. The aerial parts were hydro-distilled using a Clevenger apparatus for 4 h, giving yellow oil in 0.9% yield. The oil was dried over anhydrous sodium sulfate and stored at 4ºC. The plant was previously identified by the Department of Botany of the Research Institute of Forests and Rangelands (TARI) in Tehran, Iran. A voucher specimen (No. 58416) has been deposited at the TARI Herbarium (14, 16).

The components of Satureja khozestanica essential oil were analyzed with gas chromatography/ mass spectrometry (GC/MS) in Research Center of Lorestan University (Table 1). The complete details of this GC/MS will be published in future.

**Table 1:** The components of *Satureja khuzestanica *essential oil that analyzed by GC/MS

**No.**	**Compound name**	**Area (%)**	**No**	**Compound name**	**Area (%)**
1	3-Methyl butanol	0.14	19	*β*-Phellandrene	0.34
2	Eugenol	1.33	20	*α *-Thujene	1.26
3	1,8-Cineole	0.24	21	*β *-Caryophyllene	0.7
4	*α *-Pinene	0.99	22	*γ*-Terpinene	2.77
5	Geranyl acetone	0.5	23	Camphene	0.14
6	cis-Sabinene hydrate	0.68	24	*α*-Franesene	0.7
7	iso-Amylpropionate	0.23	25	Terpinolene	0.22
8	*β*-Bisabolene	3.77	26	*β*-Pinene	0.32
9	Linalool	3.32	27	*α*-Bisabolene	0.51
10	Myrcene	2.43	28	Nonanal	0.24
11	Caryophyllene oxide	1.53	29	trans-2-Carene-4-ol	0.73
12	4-Terpineol	4.1	30	*β*-Udesmol	0.32
13	iso-Butyl-2-methyl butyrate	0.19	31	*α*-Terpineol	0.42
14	Heptadecane	0.19	32	3-Carene	0.36
15	Thymyl methyl ether	1.21	33	*α*-Bisabolol	0.27
16	*α-*Terpinene	0.73	34	trans-Dihydrocarvone	0.26
17	Musk ambrette	0.08	35	para-Cymene	5.61

*Animals*

Thirty male mature Sprague-Dawley rats (180-200 g) were obtained from Pasteur Institute of Tehran and were allowed to adapt themselves with the new location for one week. This study was approved by the Animal Ethics Committee of the Medical University of Lorestan with accordance to the national health and medical research council guidelines. The rats were divided to tree groups (10 per each). The studied groups were as follows: group 1 as control, group 2 as diabetic without treatment and the 3rd group as diabetic treatment with .SKE

*Diabetes induction*

Diabetes was induced after overnight fasting in the second and third groups by injection of alloxan monohydrate (120 mg/Kg) subcutaneously (17). Beta-cell degradation by alloxan leads to release of more insulin. Owing to acute hypoglycemia, the rats received 10% sucrose solution for 48 h instead of drinking water. Five days after induction of diabetes, blood samples were gathered from the end part of tails. Blood glucose was measured by glucometer and the rats with blood glucose level of ≥ 300 mg/dL (16.7 mmol/L) were considered as diabetic (18). During the first five days after diabetes induction, 1-3 rats per group died because of alloxan toxicity. The rats were kept at 12/12 dark-light period in 21 ± 3°C temperature. All animals were allowed free access to food and water *ad libitum *during the experiment.

The third group was treated with SKE by 500 ppm in drinking water, respectively for eight weeks (16). The treatment was begun at the first day of diabetes induction. After 8 weeks of treatment, animals were anesthetized (Nesdonal 50 mg/Kg, IP), blood samples were obtained from hearts and allowed to clot for 20 min in laboratory temperature and then centrifuged at 2000 rpm for 10 min for serum separation (14).

*Biochemical study*

The serum levels of fasting blood glucose (FBG), triglyceride (TG), cholesterol (C), low density lipoprotein (LDL), very low density lipoprotein (VLDL), high density lipoprotein (HDL), atherogenic index and the activities of alanine aminotransferase (ALT), aspartate aminotransferase (AST) and alkaline phosphatase (ALP) of all groups were analyzed.

FBG, Cholesterol and triglyceride concentrations and ALT, AST and ALP activity were measured via biochemical analyzer using commercial kits (Olympus AU-600, Tokyo, Japan). HDL was measured in the supernatant after the precipitation of the Apo-B containing lipoproteins (LDL and VLDL) using polyanions in the presence of a divalent cation (18). LDL and VLDL were determined by calculation using the Freidewald equation (20, 21).

*Total antioxidant activity*

Total antioxidant activity of the test samples was determinated according to the method of prrieto *et al. *In brief, 0.3 mL of the sample was mixed with 3.0 mL of reagent solution (0.6 M sulfuric acid, 28 mM sodium phosphate and 4 mM ammonium molybdate). Reaction mixture was incubated at 95ºC for 90 min under water bath. Absorbance of all the sample mixtures was measured at 695 nm. The total antioxidant activity was expressed as the number of equivalents of ascorbic acid acid (μmol g−1) (22).

*Statistical analysis*

All values are expressed as mean ± SEM. The data were compared between groups by Mann-whitney U-test. Statistical analyses were performed using the SPSS 13 for windows software. A p-value < 0.05 was considered statistically significant.

## Results and Discussion

*Effect of SKE on serum lipid profile and atherogenic index*

Diabetes significantly increased serum FBG, TG, Cholesterol, VLDL and LDL concentrations in comparison with the control group. Treatment of diabetic animals with SKE significantly inhibited an increase in serum FBG, TG, Cholesterol, VLDL and LDL concentrations and atherogenic index in comparison with the untreated diabetic animals. The treatment of diabetic animals with SKE also significantly inhibited decrease of serum HDL concentrations in comparison with the untreated diabetic animals (p < 0.05) (Table 1). There are reports that several medicinal plant such as Cassia auriculata flowers (23), ginger rhizome (Zingiber officinale) (24) and fruits of Musa AAA (Chenkadali) (25) have hypolipidemic effects. What’s more, there are reports that natural antioxidant such as lycopene and Natural phenolic compounds have hypolipidemic effects (26-28). Therefore medicinal plant and natural antioxidant with hypolipidemic effects could prevent or be helpful in reducing the complications of lipid profile seen in diabetic patients. The mechanism of hypolipedemic and antiatherogenic action of medicinal plant may be due to the inhibition of dietary lipid absorption in the intestine or its production by liver or stimulation of the biliary secretion of cholesterol and cholesterol excretion in the faeces (29, 30). The mechanism of hypolipedemic and antiatherogenic action of medicinal plant may also be due to the inhibition of glycation lipoproteins, enzymes and proteins that involve lipid and lipoprotein metabolism (31).

*Effect of SKE on serum ALT, AST and ALP activity*

**Figure 1:**
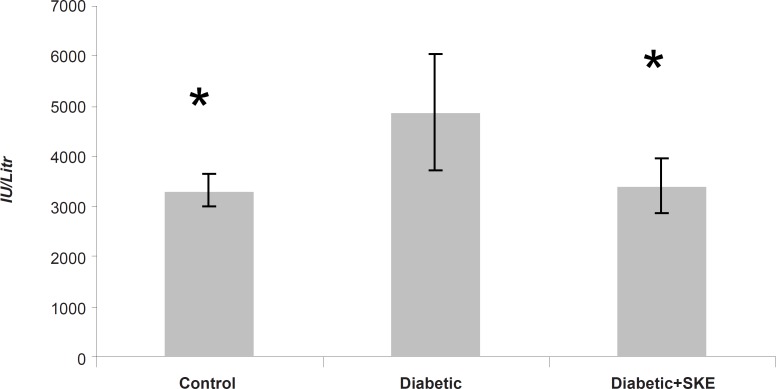
The effects of 500 ppm *Satureja khuzestanica *essential oil on serum ALP in alloxan induced diabetic rats. *Significant change in comparison with diabetic without treatment at p < 0.05

**Figure 2:**
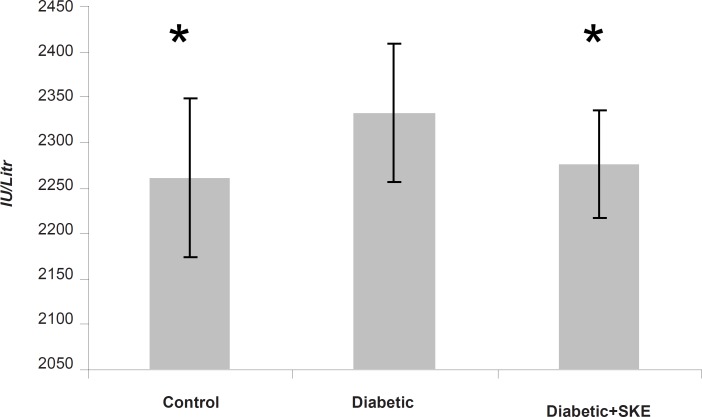
The effects of 500 ppm *Satureja khuzestanica *essential oil on seruml ALT in alloxan induced diabetic rats. *Significant change in comparison with diabetic without treatment at p < 0.05.

**Figure 3:**
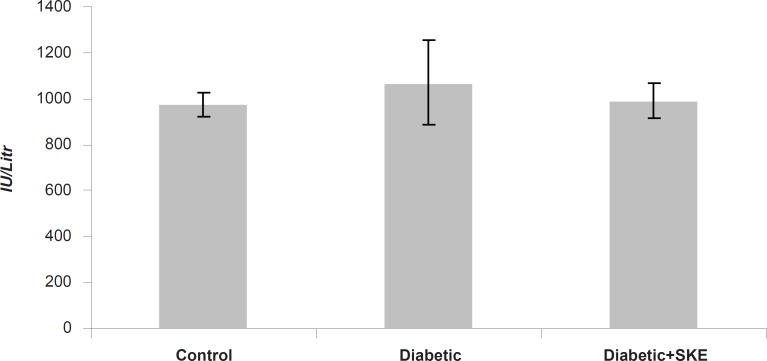
The effects of 500 ppm *Satureja khuzestanica *essential oil on serum AST in alloxan induced diabetic rats

Serum ALT, AST and ALP activity as markers of liver function significantly (p < 0.05) were increased in the untreated diabetic animals in comparison with the control group. Treatment of the diabetic animals with SKE could significantly inhibit an increase of serum ALT and ALP activity in comparison with the untreated diabetic animals. Treatment by SKE could maintain serum ALT and ALP activity of the treated animal at the same level as that of the control group (Figures 1 and 2). AST activity remained unaltered (Figure 3). ALT, AST and ALP are considered to be biochemical markers for assessing liver function (32, 33). Hepatotoxicity is evidenced by an elevation of the serum marker enzymes (33). There are reports indicating that several medicinal plants such as Agyanom mixture, Bolex bitters and Remedia mixture can reduce these liver enzymes markers (34, 35). There are also reports that natural antioxidant such as coenzyme Q_10 _and melatonin reduces these liver enzymes markers. Therefore medicinal plant and natural antioxidant with hepatoprotective action could prevent or be helpful in reducing the complications of hepatic damage seen in diabetes patients (36, 37).

*Total antioxidant activity of SKE*

**Figure 4:**
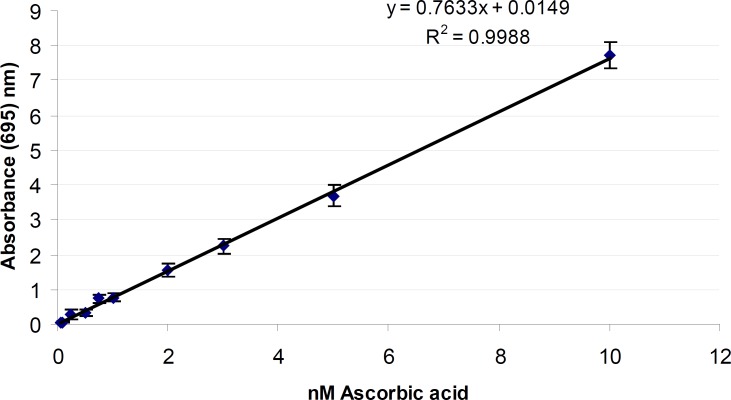
Total antioxidant activity Free radical of Ascorbic acid as standard measured in phosphomolybdenum method. Each point represents the mean of five experiments

The phosphomolybdenum method has been widely used in the assessment of total antioxidant activity of plant extracts, natural compounds and foods. Figure 4 shows the total antioxidant activity of Ascorbic acid as standard. The total antioxidant activity of SKE was 3.20 ± 0.40 nmol of ascorbic acid equivalents/g SKE. The difference in the amount of antioxidant of extracts may be attributed to the differences in the amount and kind of existing antioxidant compounds in them such as carotenoids, phenol and ascorbic acid (38). The antioxidant activity has been shown by the SKE may be due to the presence of carvacrol, tannins, triterpenoids, steroids and flavoniods (16, 41). Carvacrol is a good antioxidant-scavengers of peroxyl radicals (42) and anti-inflammatory property (43).

Our recent results indicated that SKE is found to possess a good antioxidant activity. Researches reported the role of oxidative stress as a central factor in the onset and progression of diabetic complications such as hyperlipemia and hepatic damage (4, 39, 40). Numerous reports together with our results have proved the efficacy of antioxidative supplements administration in the prevention of diabetic complications. Antioxidant therapy is used as one of the most important treatment strategies for diabetic patients for the prevention and slowing of diabetic complications progression such as hyperlipemia, hepatic damage. Moreover; beneficial effects of SKE as antioxidant, antidiabetic, anti-inflammatory and anti-hyperlipidemia, toxicity and terato-genicity tests were also performed and confirmed plant’s safety (19); moreover, this extract can be produced in large amount and low cost. For the safety of SKE, a polyphenolic compound with antioxidant and anti-inflammatory properties, consumption of SKE is introduced to diabetic patients.

**Table 2:** The effects of 500 ppm *Satureja khuzestanica *essential oil on TC, TG, LDL-C, HDL-C, VLDL-C and atherogenic index in diabetic rats

**Parameter**	**Control**	**Diabetic**	**Diabetic + SKE**
**FBG (mg/dL)**	81 ± 28*	365 ± 64	287 ± 47*#
**TG (mg/dL)**	67.00 ± 16.66*	102.00 ± 25.01	72.00 ± 22.78*
**TC (mg/dL)**	71.00 ± 16.10*	118.00 ± 25.14	87.83 ± 24.14*#
**HDL (mg/dL)**	34.66 ± 8.90*	29.75 ± 10.90	33.54 ± 9.64*
**LDL (mg/dL)**	19.60 ± 3.87*	70.85 ± 12.24	38.43 ± 9.94*#
**VLDL (mg/dL)**	13.40 ± 3.33*	20.40 ± 5.00	14.40 ± 4.56*
**Atherogenic index (units)**			
**TC/HDL-C**	2.05 ± 0.54*	3.97 ± 0.75	2.62 ± 0.62*
**LDL/HDL-C**	0.57 ± 0.08*	2.38 ± 0.67	1.15 ± 0.27*#

Values are represented as mean ± SEM SKE: *Satureja Khuzestanica *essential oil.

*Significant change in comparison with diabetic without treatment at p < 0.05.

#Significant change in comparison with control at p < 0.05.

## Conclusion

This study showed that SKE is found to posses a good antioxidant activity and has beneficial effects in reducing the elevated serum lipid profile, atherogenic index and liver enzyme markers of STZ-induced-diabetic rats. Hence, attenuation of lipid profile, atherogenic index and liver enzyme markers can decrease the risk of cardiovascular death and hepatic damage in diabetic patients. 
